# A Reference Proteomic Database of *Lactobacillus plantarum* CMCC-P0002

**DOI:** 10.1371/journal.pone.0025596

**Published:** 2011-10-05

**Authors:** Li Zhu, Wei Hu, Datao Liu, Wanhong Tian, Gang Yu, Xiankai Liu, Jie Wang, Erling Feng, Xuemin Zhang, Bei Chen, Ming Zeng, Hengliang Wang

**Affiliations:** 1 State Key Laboratory of Pathogen and Biosecurity, Beijing Institute of Biotechnology, Beijing, China; 2 National Institute for the Control of Pharmaceutical and Biological Products, Beijing, China; 3 Sine Pharmaceutical Corp. Ltd., Shanghai, China; 4 National Center of Biomedical Analysis, Beijing, China; Russian Academy of Sciences, Institute for Biological Instrumentation, Russian Federation

## Abstract

*Lactobacillus plantarum* is a widespread probiotic bacteria found in many fermented food products. In this study, the whole-cell proteins and secretory proteins of *L. plantarum* were separated by two-dimensional electrophoresis method. A total of 434 proteins were identified by tandem mass spectrometry, including a plasmid-encoded hypothetical protein pLP9000_05. The information of first 20 highest abundance proteins was listed for the further genetic manipulation of *L. plantarum*, such as construction of high-level expressions system. Furthermore, the first interaction map of *L. plantarum* was established by Blue-Native/SDS-PAGE technique. A heterodimeric complex composed of maltose phosphorylase Map3 and Map2, and two homodimeric complexes composed of Map3 and Map2 respectively, were identified at the same time, indicating the important roles of these proteins. These findings provided valuable information for the further proteomic researches of *L. plantarum*.

## Introduction

*Lactobacillus plantarum* is a kind of beneficial lactic acid bacteria (LAB) widely used by the food industries now. It can balance the gastrointestinal tract, and inhibit the growth of pathogenic bacteria through competition for nutrients, while also promoting a healthy immune system [1]. Thus, supplements of *L. plantarum*, as with most probiotics, are now available at health food stores. It has been used in the treatment for Irritable Bowel Syndrome [2]. Furthermore, *L. plantarum* has been explored as one of the most safe, effective mucosal delivery vehicles for vaccines and therapeutic molecules [3]. This is an exciting and promising research area for the preparation of oral vaccines.

Till now, the full genomes of three *L. plantarum* strains have been sequenced, including *L. plantarum* WCFS1 [4], *L. plantarum* JDM1 [5] and *L. plantarum* ST-III [6]. Analysis revealed that this species had one of the largest genomes known among lactic acid bacteria, and they all contained one or more plasmids. While the genomes may be the blueprint for an organism, proteins represent the actual functional molecules required by all life processes in the cell. Investigations at the proteomic level can provide insights into protein abundance and some information about protein post-translational modifications, which are the crucial complement and verification for genome annotations.

Recently, more and more proteomic studies focused on *L. plantarum* have been reported. Cohen *et al.* established the first reference proteome map of cytosolic proteins of *L. plantarum* and analyzed the dynamic proteomic changes during transition from log to stationary growth [7]. Cell surface-associated proteins were separated and identified by another group [8]. Key proteins in the adhesion of *L. plantarum*, and in the response to tannic acid, bile, and alkaline stress were also analyzed by proteomic methods [9], [10], [11], [12], [13]. These proteomic studies provide valuable data and pave the way for a more comprehensive insight into the molecular basis of *L. plantarum*.

However, the proteins identified in all of these studies are very limited. Even in the reference database, there are only 123 proteins were listed, corresponding to about 3.3% coverage of the genome [7], while this number is about 21.4% (369 proteins) in *Bifidobacterium longum* [14], another beneficial lactic acid bacterium. Due to the rapid development and optimization of two-dimensional polyacrylamide gel electrophoresis (2-DE) method, the resolution and sensitivity of today's 2-DE technique has been greatly improved. More proteins need to be identified as a reference for further proteomics research of *L. plantarum*, particularly for comparative studies.

## Results and Discussion

### 1 Proteome of *L. plantarum* CMCC-P0002

#### 1.1 2-DE maps of whole-cell proteins of *L. plantarum*

**Figure 1:**
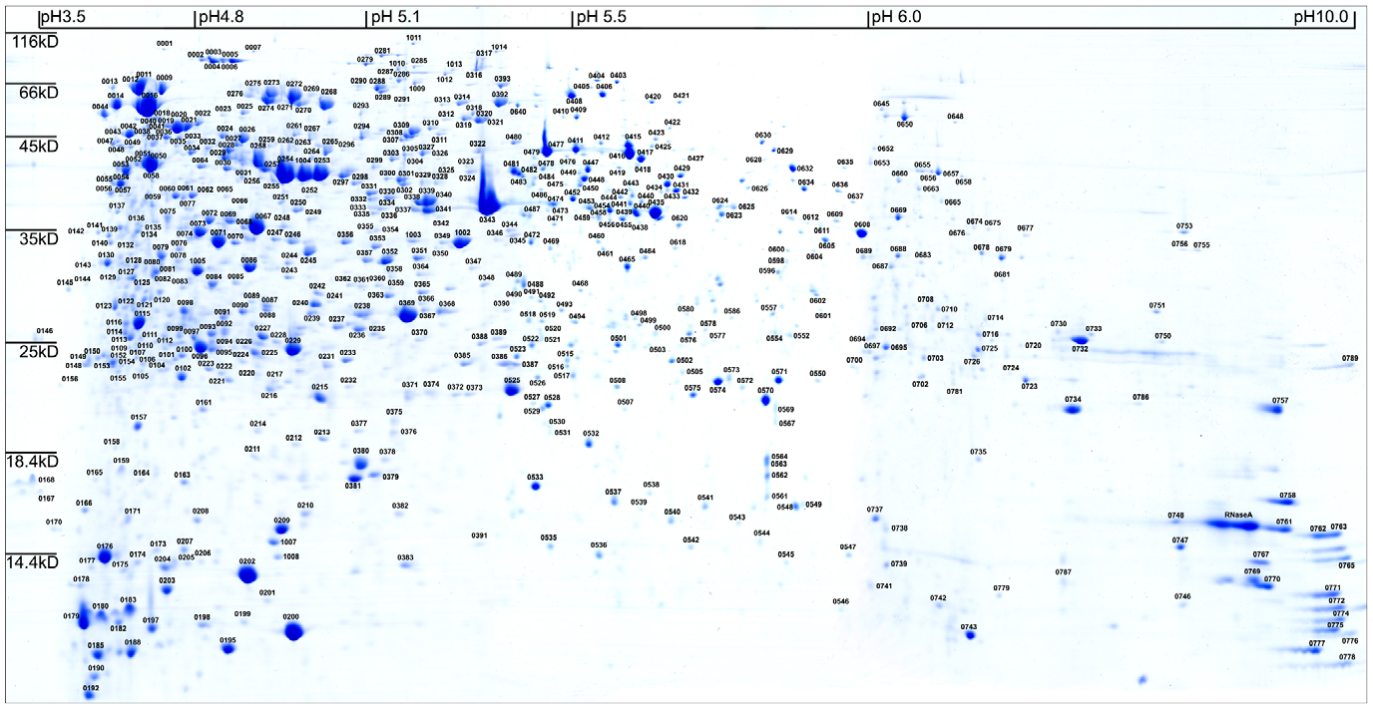
2-DE reference map of whole-cell proteins of *L. plantarum*. The identified spots were labeled on the integrated 2-DE map of pH 3–5.6 NL, pH 5.5–6.7 and pH 6–11.

To get a global view of the distribution of protein spots, an IPG strip with pH range 3–11 was first used in the pre-experiment. The results showed that most protein spots were scattered in the isoelectric point (p*I*) range of pH 4–7. So, IPG strips of pH 3–5.6 NL and pH 5.5–6.7 were used to resolve protein in the densely populated pH 4.0–7.0 zone. Those basic proteins were also analyzed using IPG strips of pH 6.0–11.0. The separate graphs of the above different pH gradient range gels were merged to produce a single artificial gel map of pH 3.0–11.0 (Figure 1).

This artificial map displayed more than 900 Coomassie-stained protein spots. After destaining and in-gel trypsin digestion, 725 spots were subjected to MALDI-TOF/TOF MS analysis. Given that the genome of our strain has not been sequenced, the acquired mass spectra were initially searched against different databases generated from all of three published *L. plantarum* genomes. The results showed that number of identified proteins was larger when genome of *L. plantarum* JDM1 was used. This indicated that *L. plantarum* CMCC-P0002 was genetically closely related to *L. plantarum* JDM1. Finally, a total of 603 spots representing 423 proteins, including 122 hypothetical proteins, were successfully identified (Table S1) when searching against the database of *L. plantarum* JDM1.

#### 1.2 2-DE map of secretory proteins of *L. plantarum*

**Figure 2:**
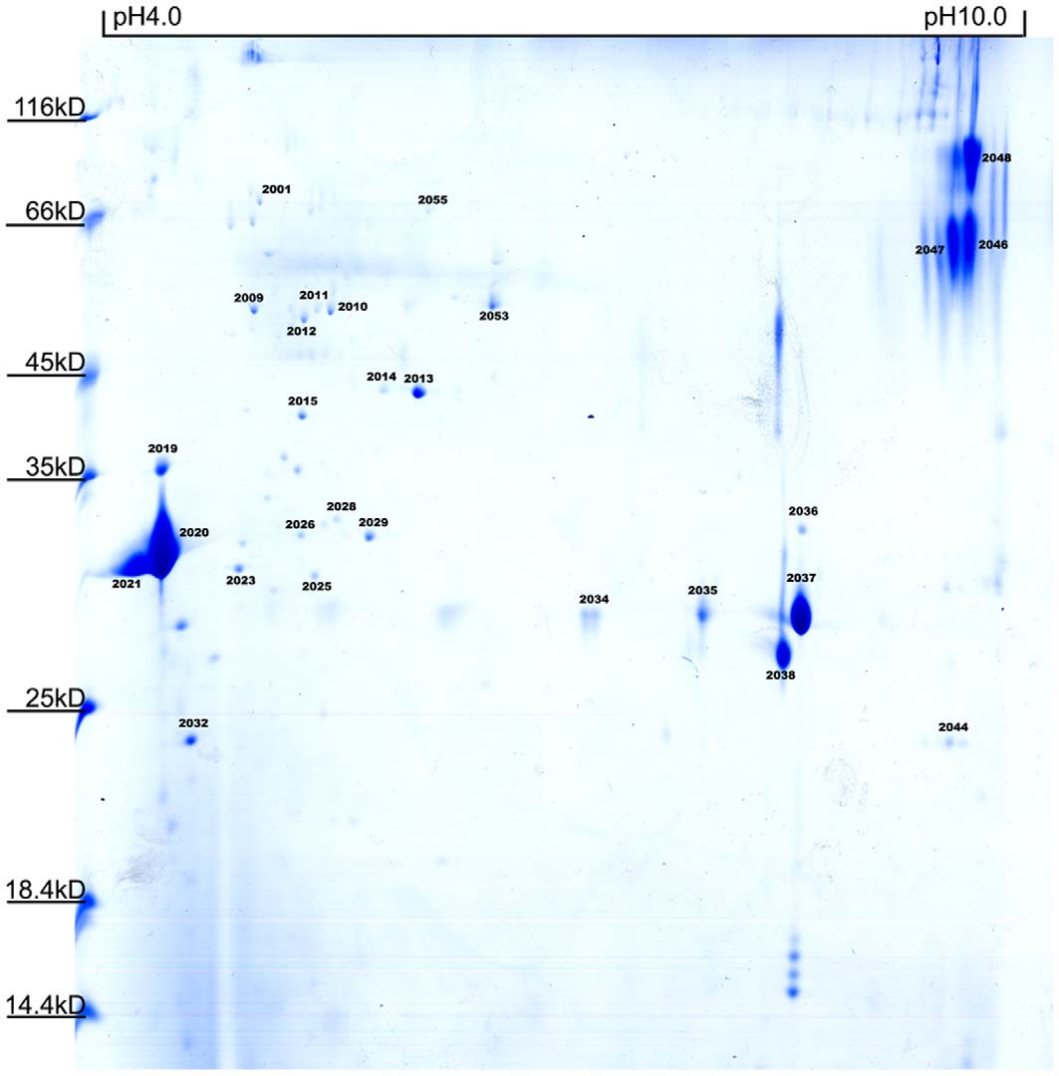
2-DE map of secretory proteins of *L. plantarum* separating by pH 3–11 IPG strips.

It has been proved that secretory proteins of lactic acid bacteria play important roles in preventing pathogen adhesion to intestinal surfaces and exchanging signals with the host. Characterization of these proteins would contribute to a better understanding of the interaction of bacteria with its host environments. Here, secretory proteins of *L. plantarum* CMCC-P0002 were also separated and analyzed (Figure 2). A total of 28 spots representing 22 proteins, including 11 proteins not detected in whole-cell maps, were successfully identified (Table S2). Among these, 12 proteins have been annotated as extracellular protein, including known “moonlighting proteins” GAPDH, enolase and EF-Tu [15]. The information of these proteins is valuable for construction of high-level secretory expressions system in *L. plantarum*.

### 2 Data Analysis

#### 2.1 Comparison with the former reference proteome database

**Figure 3:**
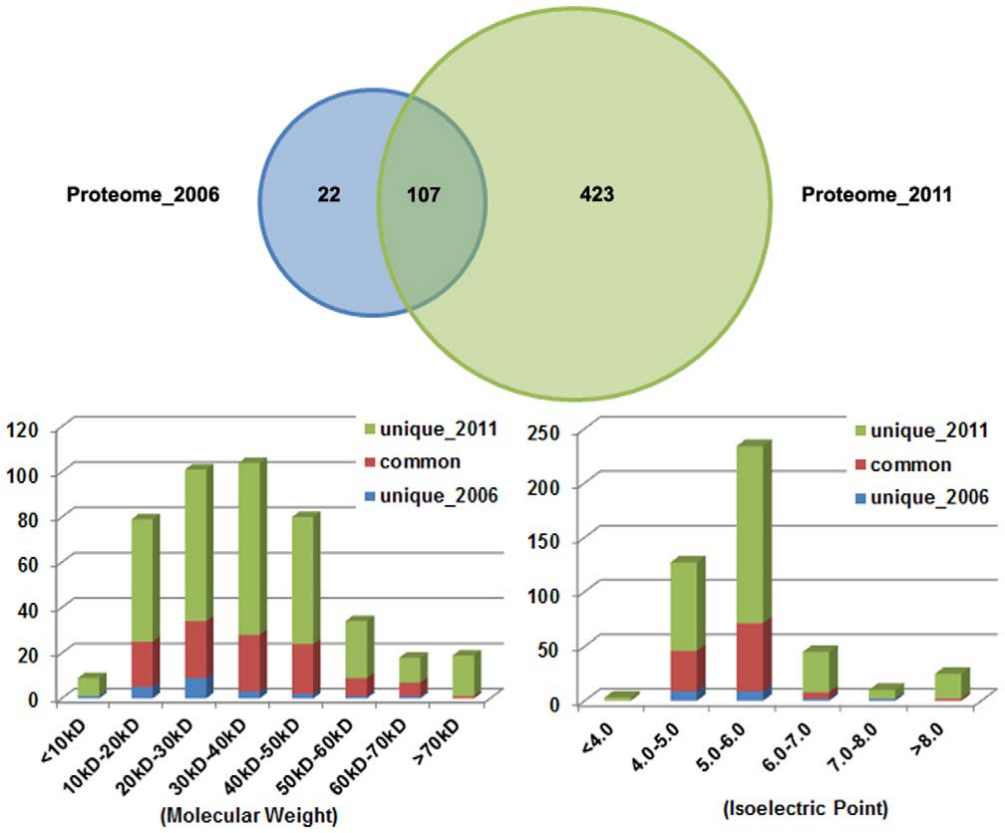
Comparisons of proteome reference databases. Venn diagram showed the numbers of unique and shared non-redundant identified proteins between two databases. Column diagram showed the distribution of identified proteins according to their theoretical MWs and p*I*s of identified proteins.

The first reference proteome map of *L. plantarum* was established in 2006 [7], with 129 proteins identified. Among these proteins, 107 proteins (about 83%) were also identified in this study. Figure 3 illustrates the degree of overlap between the former dataset and our report. The distributions of unique and shared non-redundant identified proteins according to their theoretical MWs and p*I*s were also shown in the figure. From the figure, we can see that there are significantly more proteins identified than previous report, particularly those proteins with higher MWs or p*I*s. More importantly, all of the protein identifications in this study are supported by at least one high-quality tandem mass spectrum, making the results more credible than PMF identification. Thus, our results are essential improvements to the reference proteome map of *L. plantarum*, and valuable for further comparative proteomic analyses.

#### 2.2 Predicted and actual proteome of *L. plantarum*

**Figure 4:**
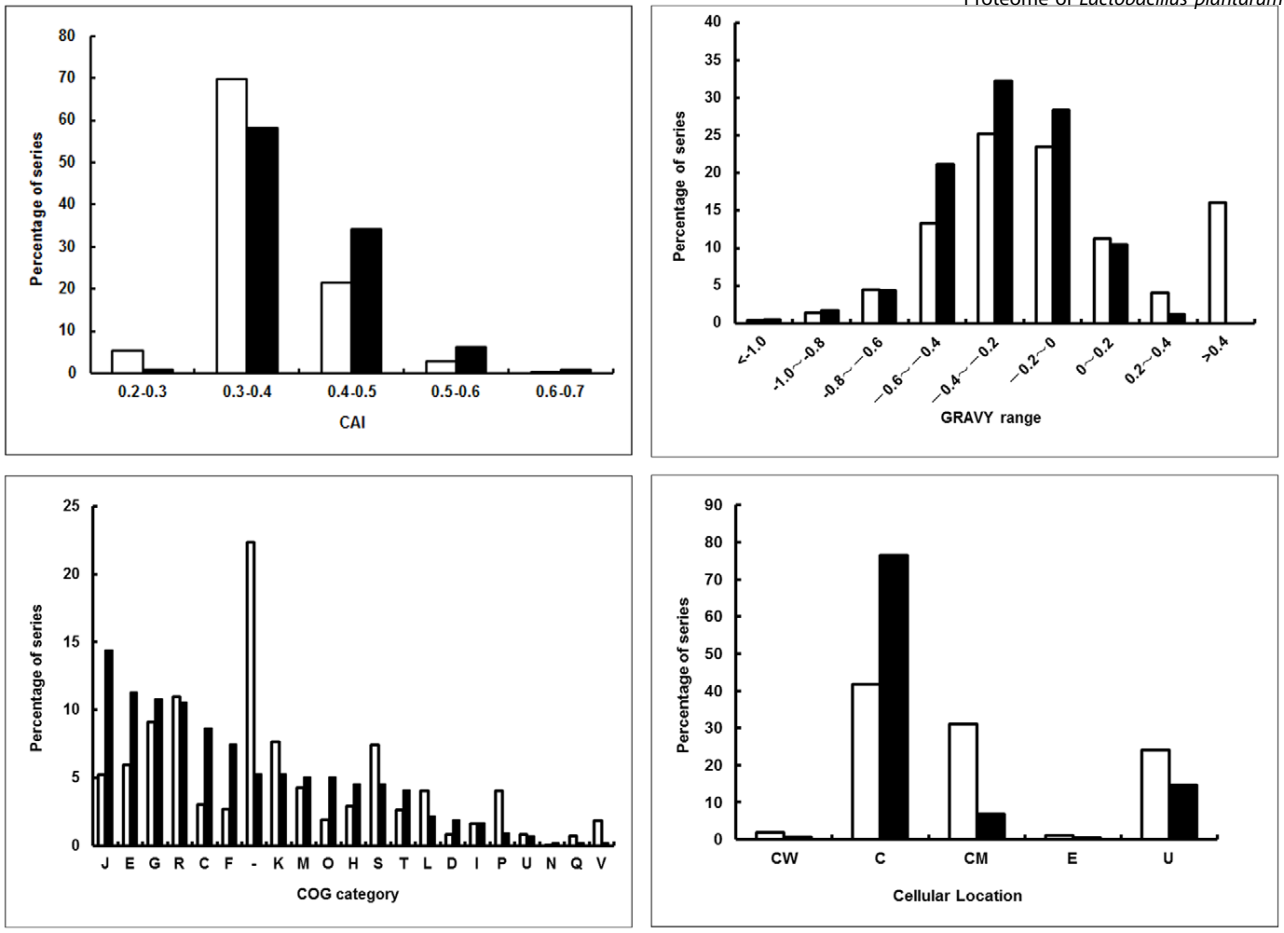
Comparative analyses of predicted and actual proteome of *L. plantarum*. Frequency distribution of the CAI, GRAVY, COGs and Cellular Locations of predicted proteome (while bars) and identified proteins (black bars).

The molecular weight (MW) and p*I* values of the protein spots on the 2-DE gels were compared with the theoretical values. As shown in Figure S1, MW and p*I* values estimated by gel electrophoresis matched closely with predicted values, except for some discrepancies. The differences in MW values seem to be more than those in p*I* values, probably due to the cleavage of signal peptides or other structural sequences. The CAI and GRAVY index distributions of genes coding for the proteins identified are also compared with those of all predicted proteins (Figure 4). Briefly, proteins encoded by genes with a low CAI and extreme GRAVY values are difficult to identify, similar to those of previous reports from *L. lactis* [16], and *B. longum* [14]. Based on COG (Clusters of Orthologous Groups) information, experimentally identified proteins were grouped into cellular roles and are summarized in Figure 4. Proteins related to translation (category J) are the category containing the most identified proteins. The cellular localizations of all identified proteins, predicted by PSORT Version 2.0 (www.psort.org), are also compared with those of all predicted proteins (Figure 4). Briefly, 318 proteins identified are cytoplasmic, 28 proteins are predicted to reside in the cytoplasmic membrane, 3 proteins are predicted to be located in the cell wall, and 2 proteins are predicted to be secretory proteins.

#### 2.2 Protein abundance

One of the advantages of 2-DE methods is that it can directly display the abundance of each protein spot. The details of first 20 highest abundance proteins in our 2-DE whole-cell maps are summarized in Table 1. Most of these proteins are related to translation, carbohydrate metabolism and chaperones. The information of protein expression abundance is very important for the further genetic manipulation of *L. plantarum*. For example, if an exogenous gene should to be introduced into *L. plantarum*, its transcriptional regulatory region can be replaced with the region upstream of above high abundance genes to achieve high-level expression.

**Table 1:** The first 20 highest abundance proteins.

Spot ID	GI	Gene	COG(S)	MW	p*I*	CAI	Score	Protein Name
0016	254044697	*groEL*	COG0459	55071	4.43	0.52	822	chaperonin GroEL
0343	254044748	*gapB*	COG0057G	37158	5.34	0.64	498	glyceraldehyde 3-phosphate dehydrogenase
0254	254045870	*tuf*	COG0050	40599	4.96	0.56	734	elongation factor Tu
0200	254044696	*groES*	COG0234	12112	4.97	0.44	334	co-chaperonin GroES
0202	254045170	*hpr*	COG1925	13887	4.89	0.47	122	phosphocarrier protein HPr
0067	254045813	*ldhD*	COG1052	35021	4.91	0.52	489	D-lactate dehydrogenase
0369	254044389	*fba*	COG0191	26941	5.18	0.59	607	fructose-bisphosphate aldolase
0179	254044655	*rplL*	COG0222	12421	3.96	0.56	477	ribosomal protein L12/L7
0050	254044751	*eno*	COG0148	41682	4.47	0.60	582	phosphopyruvate hydratase
0253	254044749	*pgk*	COG0126	40453	5.02	0.57	507	phosphoglycerate kinase
0176	254044223	*hsp1*	COG0071	14514	4.12	0.41	505	small heat shock protein
0011	254045785	*dnaK*	COG0443	62633	4.38	0.43	531	molecular chaperone DnaK
0229	254046628	*gpmA*	COG0588	24407	4.97	0.52	661	phosphoglyceromutase
0435	254044265	*msmK1*	COG3839	36541	5.64	0.43	361	multiple sugar ABC transporter, ATP-binding protein
0071	254044532	*ldhL1*	COG0039	33542	4.84	0.52	483	L-lactate dehydrogenase
0258	254046108	*pgi*	COG0166	41907	4.91	0.48	509	glucose-6-phosphate isomerase
0525	254044704	*-*	COG1544	21982	5.38	0.43	554	ribosomal protein S30EA
1002	254045811	*rpsB*	COG0052	33391	5.28	0.49	454	30S ribosomal protein S2
0115	254044750	*tpiA*	COG0149	26391	4.37	0.58	528	triosephosphate isomerase
0096	254044171	*pgmB2*	COG0637	24603	4.81	0.37	441	beta-phosphoglucomutase

An interesting finding should also be noted that one hypothetical protein encoded by the plasmid pLP9000 was identified in this study. The abundance of this protein (pLP9000_05, spot ID 0533) is not low, indicating that it might play an important role in the metabolism of *L. plantarum*. More investigations should be performed to reveal its functions.

### 3 Blue-Native/SDS-PAGE analysis of soluble protein complexes of *L. plantarum*

**Figure 5:**
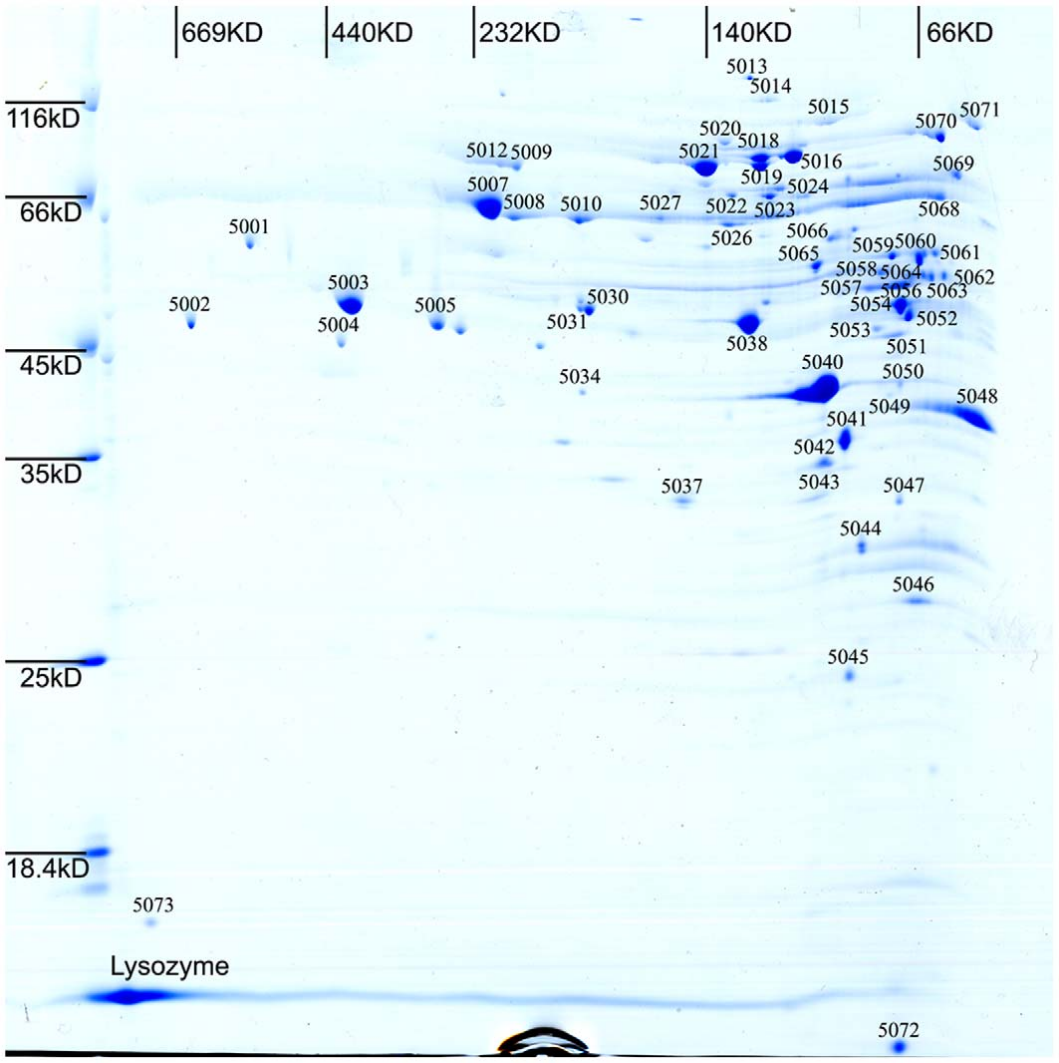
BN/SDS-PAGE map of the *L. plantarum*.

In living cells, interactions with other proteins are very important for a majority of proteins to carry out their biological functions. Therefore, it seems to be more valuable if we could separate and identify the components of protein complexes in cells at a global level. Another two dimensional electrophoretic technique, Blue-Native/SDS-PAGE, is the most convenient and robust method to generate large-scale protein-protein interaction maps. In this method, the protein complexes will initially be separated in non-denaturing conditions to maintain, for the most part, their interactions and structures as they would be in the cell. And then, the gel lane will be cut off for SDS-PAGE to separate all components of complexes. We applied this method in this study, and got the first interaction map of *L. plantarum* (Figure 5). After destaining, trypsin digestion and MS analysis, a total of 55 spots representing 49 proteins were successfully identified (Table S3).

As shown in Figure 4, protein Map3 (spot ID 5018) and Map2 (spot ID 5019) were arranged in a straight line in the gel, suggesting that these two proteins could form a heterodimeric complex. Interestingly, Map3 (spot ID 5016) and Map2 (spot ID 5021) were also respectively identified as homodimeric complexes at different positions of the same map. This phenomenon indicated that the structures and functions of Map3 and Map2 were so similar that they could substitute each other though they had different MWs. These two proteins are annotated as maltose phosphorylase in the genome [5]. But it's still not clear why these two isoenzymes were expressed at the same time to form three different complexes.

## Materials and Methods

### Bacterial strains and growth conditions

*L. plantarum* CMCC-P0002 was obtainded from the China Medical Culture Collection Centre (CMCC). Bacteria were cultured in 10 mL Man-Rogosa-Sharp (MRS) medium under anaerobic conditions at 37°C overnight. Then, the overnight culture were transferred to 400 mL MRS medium to make a 1∶100 dilution, and cultured at 37°C under anaerobic conditions. Bacteria were harvested in the stationary phase (after 17 h, OD_600 nm_ = 8.0).

### Preparation of whole-cell protein extracts

The preparation of whole cell protein extracts was performed as described previously [14]. The protein concentration of samples was measured using the PlusOne 2-D Quant Kit (GE healthcare, USA), and 0.8 mg aliquots were stored at −80°C.

### Preparation of secretory proteins

*L. plantarum* CMCC-P0002 was cultured as described above. Cells were removed by centrifugation at 4°C for 10 min at 10 000×g and the supernatant was transferred to a sterile tube and filtered (0.22 µm). 100 mL acetone containing 0.1% DTT precooling at −20°C was added to same amount of supernatant, then vortexed for 1 min and placed at −20°C overnight. This mixture solution was centrifuged (10 000×g) for 30 min at 4°C, and the supernatant was discarded. The pellet was washed with precooling acetone containing 0.1% DTT four times. The pellet was dried at room temperature, then pellet was resuspended 1 mL lysate solution (7 M urea, 2 M thiourea, 1% DTT, and 4% CHAPS), and its protein concentration was determined using the PlusOne 2-D Quant Kit (GE Healthcare). The supernatant was stored in 200 µg aliquots at −80°C.

### Preparation of protein complexes

*L. plantarum* CMCC-P0002 was cultured as described above. Cells were harvested by centrifugation at 4°C for 10 min at 10 000×g and the pellet was washed once with cold phosphate-buffered saline (PBS), and resuspended in lysis buffer (20 mM Tris-HCl, 137 mM NaCl, 2 mM pH 8.0 EDTA, 10% glycerol, adjust pH at 7.4 and stored at 4°C. Add 1 mg/ml lysozyme, 1000 unit DNase I, 0.1% Triton X-100 and protease inhibitor before use). The solution was incubated at 37°C for 2.5 h. Cell debris was removed by centrifugation at 20, 000 *g* for 20 min. The protein concentration of samples was measured using the PlusOne 2-D Quant Kit (GE healthcare, USA).

### Two-dimensional Polyacrylamide Gel Electrophoresis (2-DE)

To obtain better separation, pH 3.0–5.6 NL, pH 5.5–6.7 and pH 6–11 immobilized pH gradient (IPG) strips (18 cm, GE healthcare, USA) were used for whole-cell protein and pH 3–11 IPG strips was used for secretory proteins in the isoelectric focusing (IEF) analysis. For each analysis, the proteins were treated with the 2-D Clean-Up Kit (GE healthcare, USA) according to the kit instructions and resuspended in 350 µl rehydration buffer [7 M Urea, 2 M thiourea, 4% CHAPS, 1% DTT, 0.5% IPG Buffer (same pH range of the IPG strip)]. The following 2D procedure and the in-gel protein digestion were carried out as described previously. Briefly, an 800 µg protein sample was used to rehydrate 18 cm immobilized gradient strip for 12 hrs at 20°C. IEF was conducted at 300 V (1 hr), 600 V (1 hr), 1000 V (1 hr), 8000 V (8 hr). The temperature was maintained at 20°C. After IEF, each strip was equilibrated for 15 min in equilibrium buffer (2% SDS, 50 mM Tris-HCl, pH 8.8, 6 M urea, 30% glycerol, 0.002% bromophenol blue and 1% DTT). For the second gels dimensional separation, each strip was overlaid onto 12.5% SDS polyacrylamide. The gels were stained using Colloidal CBB (0.1% CBB G-250, 34% methanol, 17% ammonium sulfate, 3% phosphoric acid) overnight.

### BN/SDS-PAGE

BN-PAGE method was carried out as described previously [17]. A linear 4%–15% acrylamide gradient gel with a 3.2% stacking gel was used for separating whole cell protein complex. Anode and cathode electrophoresis buffers were the same as described by Mahima Swamy *et al* [18]. Cathode buffer was supplemented with 0.02% (w/v) Coomassie brilliant blue G-250 when necessary. Before loading, 1∶5(v/v) sample loading buffer [750 mmol/L 6-amino-n-caproic acid, 5% w/v Coomassie brilliant blue G-250 and 20% (v/v) glycerol] was added to the sample. The gel was initially run at 100 V for 1 h at 4°C. Then, the gel was run at 300 V for 16 h. After the first dimensional electrophoresis, the lane was cut off from the gel and equilibrated. SDS-PAGE was performed using a 12.5% separating gel according to standard protocols.

### Image analysis and in-gel protein digestion

Image analysis was processed by ImageMaster 2D Platinum software (GE healthcare). To facilitate the discrimination between real spots and artifacts, the spot detection parameters were adjusted as follows: smooth 3, min area 50, and saliency 6. The relative volume of each spot was determined from the spot intensities in pixel units and normalized to the sum of the intensities of all the spots on the gel. The protein spots were carefully excised from the CBB G-250 stained 2-DE gel, destained, washed, and then digested for 13 hrs with sequencing grade modified trypsin (Roche, USA). Peptides from digested proteins were used for MALDI-TOF/TOF analysis.

### MALDI-TOF/TOF MS

MALDI-TOF/TOF MS measurements were performed on a Bruker Ultraflex III MALDI-TOF/TOF MS (Bruker Daltonics, Germany) operating in reflectron mode with 20 kV accelerating voltage and 23 kV reflecting voltage. A saturated solution of α-cyano-4-hydroxycinnamic acid in 50% acetonitrile and 0.1% trifluoroacetic acid was used as the matrix. One microliter of the matrix solution and sample solution at a ratio of 1∶1 was applied onto the Score384 target well. Series of eight samples are spotted around one external calibration mixture. The SNAP algorithm in FlexAnalysis™ 3.4 was used to pick up the peaks in the mass range m/z 1000–5000. The subsequent MS/MS analysis was performed in a data-dependent manner, and the 5 most abundant ions were subjected to high energy CID analysis. The collision energy was set to 1 keV, and nitrogen was used as the collision gas.

### Data Interpretation and Database Searching

The MS/MS results were searched by the program Mascot 2.1 (Matrix Science Ltd) against the database containing *L. plantarum* JDM1 (GI: 254044096), the plasmid pLP2000 (GI: 20853777) and pLP9000 (GI: 20853804). The searching results were checked against the NCBInr database. The search parameters are as following: trypsin digestion with one missed cleavage; carbamidomethyl modification of cysteine as a fixed modification and oxidation of methionine as a variable modification; peptide tolerance maximum, ±0.2 Da; MS/MS tolerance maximum, ±0.6 Da; peptide charge, +1; monoisotopic mass. Scores greater than 49 are significant (p<0.05) for a local Peptide Mass Fingerprinting (PMF) search. Ion scores greater than 20 are significant (p<0.05) for a local MS/MS search.

## Supporting Information

Figure S1 Representation of 2-D gel separation of the proteome according to predicted (left) and identified (right) p*I* and MW.
(TIF)

Table S1 The detailed information of whole-cell proteins identified in this work.
(XLS)

Table S2 The detailed information of secretory proteins identified in this work.
(XLS)

Table S3 The detailed information of components of protein complexes identified in this work.
(XLS)
