# Supplementary figures and images for: A Reference Proteomic Database of Lactobacillus plantarum CMCC-P0002
Source: PLoS One. 2011 Oct 5;6(10):e25596. doi: 10.1371/journal.pone.0025596 (PMC3187783; doi:10.1371/journal.pone.0025596)

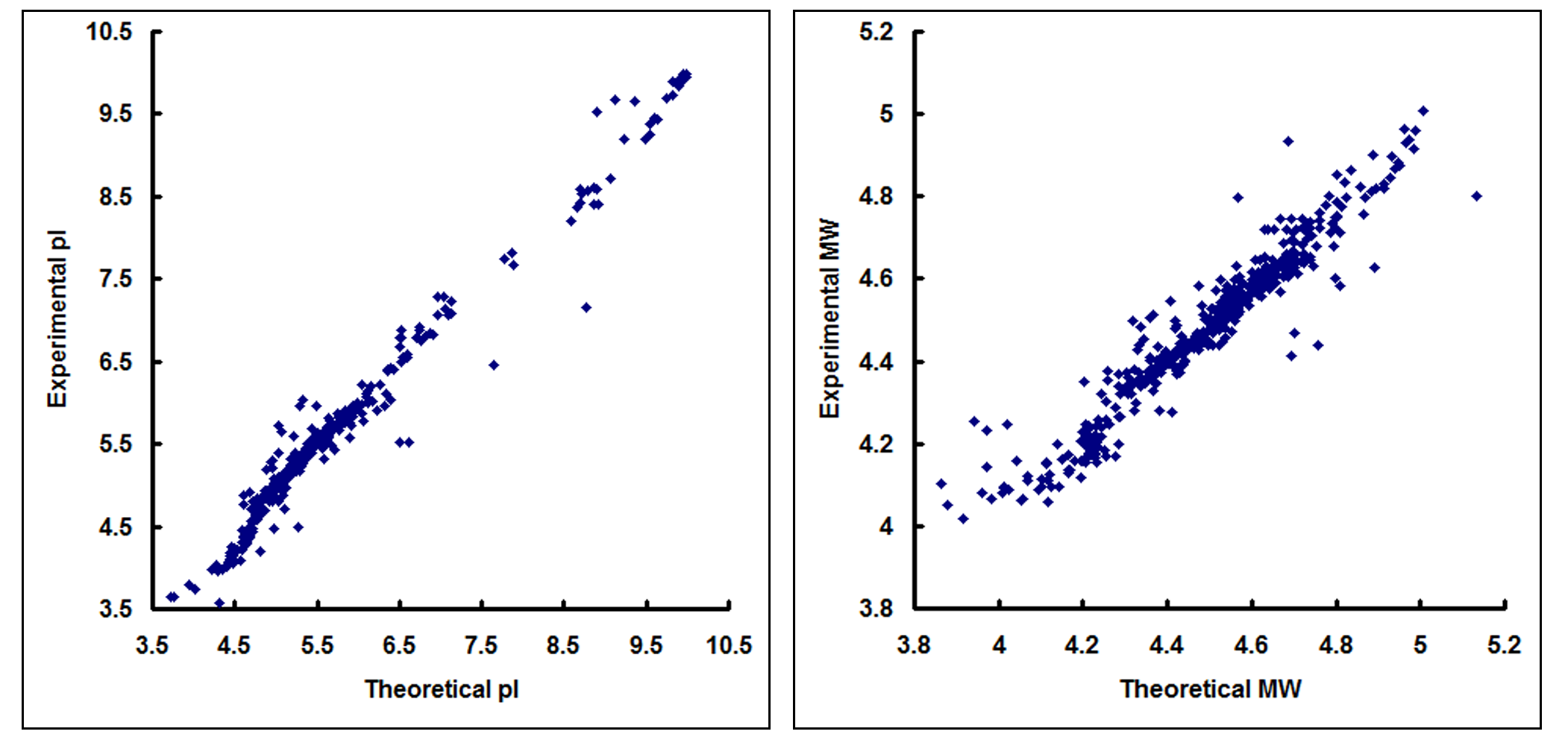

Supplement: Figure S1 — Representation of 2-D gel separation of the proteome according to predicted (left) and identified (right) pI and MW. (TIF) [file pone.0025596.s001.tif]
